# High throughput embryonic zebrafish test with automated dechorionation to evaluate nanomaterial toxicity

**DOI:** 10.1371/journal.pone.0274011

**Published:** 2022-09-16

**Authors:** Chance M. Carbaugh, William H. van der Schalie, Mark W. Widder

**Affiliations:** 1 Walter Reed Army Institute of Research, Silver Springs, Maryland, United States of America; 2 Oak Ridge Institute for Science Technology, Oak Ridge, Tennessee, United States of America; 3 General Dynamics Information Technology, Falls Church, Virginia, United States of America; Oregon State University, UNITED STATES

## Abstract

Engineered nanomaterials pose occupational health and environmental concerns as they possess unique physical and chemical properties that can contribute to toxicity. High throughput toxicity screening methods are needed to address the increasing number of nanomaterials in production. Here we used a zebrafish photomotor response (PMR) test to evaluate a set of fifteen nanomaterials with military relevance. Automated dechorionation of zebrafish embryos was used to enhance nanomaterials bioavailability. Optimal PMR activity in zebrafish embryos was found at 30–31 hours post-fertilization (hpf). Behavioral and toxicological responses were measured at 30 and 120 hpf; behavioral responses were found for thirteen of the fifteen nanomaterials and acute toxicity (LC50) levels for nine of the fifteen nanomaterials below the maximum test concentration of 500 μg/ml. Physico-chemical characterization of the nanomaterials detected endotoxin and bacterial contamination in two of the tested samples, which may have contributed to observed toxicity and reinforces the need for physical and chemical characterization of nanomaterials use in toxicity testing. The zebrafish PMR test, together with automated dechorionation, provides an initial rapid assessment of the behavioral effects and toxicity of engineered nanomaterials that can be followed up by physico-chemical characterization if toxicity is detected, reducing the amount of time and monetary constraints of physico-chemical testing.

## Introduction

Nanomaterials pose occupational health and environmental concerns as they possess unique physical and chemical properties that can contribute to toxicity. In the military, manufactured nanomaterials are used in a wide variety of applications, from smokes and obscurants to various textiles and fibers [1–3]. As the production and diverse applications of nanomaterials increases, so does the demand for rapid toxicological assessment to ensure the safety of use of such materials. Zebrafish have a number of advantages that facilitate their use for toxicity screening studies. Zebrafish models are widely used to evaluate the potential toxicity of chemicals, including the toxicological evaluation of nanomaterials [4–7]. Zebrafish husbandry requirements are low and they have a high fecundity rate; a single female zebrafish can produce in excess of one hundred embryos from a single spawning event, which facilitates zebrafish use in high throughput toxicity assays [8–11].

One such high throughput assay involves the zebrafish photomotor response (PMR). The PMR is a non-visual behavior triggered by the stimulation of photoreceptors located in the hindbrain of the developing zebrafish embryo in response to high intensity white light stimulus [12]. The PMR is a useful assay in the detection of neuroactive substances [13–15]. The efficiency of the PMR test for nanomaterials can be increased through the use of an automated dechorionation process (modified from the methods of Mandrell et al., 2012 [16]), which removes the chorion from the zebrafish embryo. This prevents the chorion from impeding the uptake of nanomaterials [17–19]. Using automated dechorionation instead of manual enzymatic dechorionation or physical dechorionation via forceps results in decreased variability (no differences in techniques between lab technicians), increased numbers of embryos that can be used for testing, and lower malformation rates [20]. In this paper, we report PMR test results with fifteen commercially available nanomaterials with military relevance that were identified as part of a risk ranking platform, the Tool for Engineered Nanomaterial Application pair Risk Ranking (TEARR). Higher-ranking nanomaterials were determined by TEARR according to the receptor type, release type, and exposure route [3]. This effort, as a proof of concept, utilized readily available TEARR-identified nanomaterials that had either potentially higher rates of human exposure or environmental impacts due to accidental or unintentional release. The zebrafish PMR model with automated dechorionation provides a high throughput approach to assess the relative toxicity of commercially available engineered nanomaterials as pre-screening to more costly and extensive physical chemical characterization. The gold standard for nanomaterial characterization required specialized equipment and is performed in sophisticated laboratories such as the NCI Nanomaterials Characterization Lab.

## Methods and materials

### Nanomaterial characterization

Nanomaterials were purchased from either nanoComposix or US Nano and were provided to the National Cancer Institute’s Nanotechnology Characterization Laboratory (NCL) for determination of their characteristics in aqueous suspensions. “The NCL evaluated sterility, endotoxin contamination and a variety of physicochemical parameters, including size/size distribution, shape, metal concentrations, purity, surface chemistry, and stability. Size and polydispersity were evaluated using transmission electron microscopy (TEM), dynamic light scattering (DLS), and asymmetric flow field-flow fractionation (AF4) coupled with DLS detection. Nanoparticle concentrations were measured using inductively coupled plasma mass spectrometry (ICP-MS) and thermogravimetric analysis (TGA). ICP-MS was also used to assess purity by detection of trace metal impurities, and stability by monitoring the level of free metal ions released in solution over time. Surface chemistry was evaluated by TGA to detect the presence of a surface coating or other excipient, and surface charge was indirectly evaluated by measuring the zeta potential. Importantly, while TGA could provide an indication of a coating and/or other excipients in the formulation, it could not identify the coatings/excipients.” [21] More details on the methods used for the physico-chemical characterization of the nanomaterials are available at: https://apps.dtic.mil/sti/pdfs/AD1098898.pdf. [21]

### Zebrafish housing and breeding

Zebrafish work was approved by the Walter Reed Army Institute of Research-Naval Medical Research Center Animal Care and Use Committee. Tubingen strain zebrafish were housed in either custom built semi-recirculating aquaculture racks or large flow through round tanks at the Walter Reed Army Institute of Research (WRAIR), an Association for Assessment and Accreditation of Laboratory Animal Care- (AAALAC-) approved facility. Overhead full spectrum LED lighting provided illumination on a 16 hour light, 8 hour dark photoperiod. Water for the aquaculture husbandry and testing facility is supplied from a mix of onsite groundwater wells and from domestic water. Domestic water is used for producing reverse osmosis (RO) permeate, which is subsequently mixed with the raw well water to produce water of appropriate hardness and alkalinity for aquaculture and testing. Water temperature was maintained within the range 25.0° C to 28.5° C. Water quality parameters were maintained within the following ranges; dissolved oxygen 60 to 100% saturation; pH 6.5 to 8.5, alkalinity 110 to 180 mg/L as CaCO_3_, hardness 150 to 210 mg/L as CaCO_3_, conductivity 400 to 1000 ohms/cm, and total ammonia less than 0.1 mg/L as NH_3_. Adult zebrafish had three daily feedings: two feedings of commercial flake food (TetraMin Tropical Flakes, Blacksburg, VA) and one feeding of live brine shrimp nauplii (Brine Shrimp Direct, Ogden, Utah), except on weekends. On weekends, two feedings were provided: one commercial flake feeding and one live brine shrimp nauplii feeding. Zebrafish breeding was performed in I-SPWAN-S breeding chambers (Techniplast, West Chester, PA) on a semi-recirculating system. Thirty adult zebrafish (6 to 12 months old and 1:1 ratio of males to females) were placed into the breeding chambers the night prior to embryo collection. Male and female zebrafish were separated by sex with a divider to ensure that no overnight spawning occurred.

### Dechorionation and embryo screening

In the morning, after a fifteen minute breeding period in the I-SPAWN-S breeding chambers, embryos were collected in glass petri dishes containing fresh fish culture water and placed into an incubator at 28.5° C for one hour post-fertilization (1 hpf) to develop to the four cell stage [22]. At this point, the embryos were screened to ensure that all embryos were at the four cell stage; any embryos not at this stage or that were not developing normally were discarded. A secondary screening was performed at 3.5 hpf to insure that only healthy, properly staged embryos would be used for testing. Between screenings, embryos were returned to the incubator. Each screening lasted only five minutes per plate to minimize developmental disruption. When the staged embryos reached 6 hpf, they were chemically dechorionated using a custom built automated dechorionator [16] by adding 83 μL of stock pronase (32 mg/mL) to approximately 500 zebrafish embryos in a glass petri dish containing 25 mL of E3 embryo media. Following the automated dechorionation process, the embryos were screened one last time to remove any embryos that still had their chorions attached or that might have been damaged by the dechorionation process. The embryos were then returned to the incubator for 30 minutes to allow them to rest before being transferred for testing.

### PMR time series test

The PMR behavioral assay consists of three phases. The first phase of the PMR is the background: from zero seconds to thirty seconds, the embryo’s spontaneous movement is recorded in the dark. Following the 30 second background period, the embryos are subjected to a flash of bright white light (18,000 lx) lasting for one second followed by nine seconds of darkness; this phase is the excitatory phase. The final phase (refractory phase) starts at 40 s and ends at 50 s. The refractory phase is initiated by a second flash of intense white light (18,000 lx) for one second followed by another nine seconds in the dark [13, 23, 24].

Before the nanomaterials were tested, a time series test was conducted to determine the time of maximum embryo response in the PMR test. The day prior to behavioral testing, seven 96 well plates (Falcon U-Bottom Tissue Culture Plates, Sterile, Corning, Corning, NY) were filled with 100 μL of MilliQ water, and one dechorionated zebrafish embryo was added per well. The plates were covered with aluminum foil and were maintained in an incubator in the dark at 28.5° C for the duration of the test, except when they were removed and uncovered (under darkroom conditions) for PMR testing. Every hour from 24 hpf through 32 hpf, PMR was determined using the Photomotor Response Analysis Tool (PRAT), a custom-built device designed and constructed by the Tanguay Lab at Oregon State University [23]. Plates had a minimum of 40 minutes rest in the darkened incubator between each PMR test to allow their photoreceptors to recover [12].

### Nanomaterial exposures

Healthy embryos were placed, one embryo per well, into a 96 well plate prefilled with 90 μL of MilliQ water using a flame polished glass pipette. The plates were then kept in the incubator at 28.5° C until the embryos reached 8 hpf. During this time, nanomaterial stock solutions were prepared in MilliQ water. Even though the nanomaterials used in this experiment were commercially prepared suspensions in water, some of the nanomaterials still settled to the bottom of the 96 well plate at higher concentrations during testing. At approximately 8 hpf, a multichannel pipette was used to dispense 10 μL of multiple concentrations into the 96 well plates. Each nanomaterial test had five chemical concentrations plus a control (N = 32 for each treatment). Control embryos were located on columns 12 and 6 on the microtiter plates. Embryos exposed to nanomaterials were located in columns 5–1 and 11–7 in increasing concentrations (2.32 μg/mL, 5 μg/mL, 10.7 μg/mL, and 50 μg/mL). The plates were covered with aluminum foil and Parafilm^®^ following the exposures. Static nanomaterial exposures ended at 120 hpf. There was no fluid replacement during exposure. The plates were removed from the incubator at 30 hpf to perform the PMR test and to collect three early developmental morphological endpoints (30hpf) and were removed a second time to collect the eighteen 120 hpf morphological endpoints [10, 15, 25] (See S1 Table for a list of morphological endpoints). After all experimental endpoints were collected the 120 hpf zebrafish embryos were euthanized by the addition of sodium hypochlorite (6.15%) to the wells of the 96 well plates. Exposures took place on multiple days across multiple weeks with different batches of dechorionated zebrafish embryos.

Exposures were repeated at either lower or higher concentrations (maximum 500 μg/mL) as needed to allow for estimations of the LC50 for each nanomaterial. Non-particulate silver (silver nitrate) was also tested for comparison to nanosilver, as it has been reported that nanosilver toxicity is likely due to dissolved ionic silver [26]. Estimates of the ionic silver fraction of the nanosilver stocks were obtained through ICP-MS conducted at the NCL.

### Data acquisition and analysis methods

Approximately 800 images were recorded for each plate during a PMR test. The camera captured images at a frame rate of 16 frames per second. These images were then combined using ImageJ (NIH, Bethesda, MD) to create an AVI file for each test. The AVI file was then converted into an mp4 utilizing a video converter. This file was then uploaded into Ethovision software (Noldus, Leesburg, VA) for processing. This processing produced an activity analysis profile per frame for each well of the 96 well plates. Any dead embryos at 24 hpf were removed from the dataset, and the remaining data were imported into Excel. The offset function in Excel was used to average the percentage of pixel change per second for each plate. These averages were then used to calculate the area under the curve (AUC) for each concentration and each phase of the PMR. The data from the AUC calculation was then reviewed, and any non-responding embryos were removed from the control groups. Non-responders were identified as embryos that had zero values across all three phases of the PMR test after the AUC calculation. The remaining AUC data set was then statistically analyzed by chemical by phase for each concentration using a 2 tailed t-test to determine statistical significance (α = 0.05). Statistically significant PMR responses were labeled as hyperactive (increased movement) or hypoactive (decrease in movement). The Trimmed Spearmen-Karber method [27] was used to generate EC50 values (morphological data) or LC50 values (mortality data).

## Results

### PMR time series test

**Fig 1:**
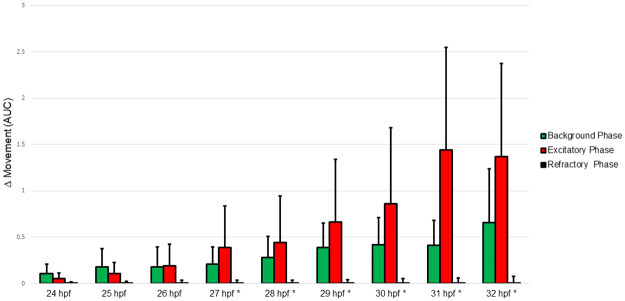
Time series test for the photomotor response of zebrafish embryos. Zebrafish photomotor response by phase. The graph depicts the control response of zebrafish embryos (n = 603) with increasing developmental age. Data generated from the PMR assay was further condensed using the area under the curve calculation by phase with standard deviation bars. Embryos that were found to be non-responders were excluded from the dataset. * = statistically significant increase in movement during the excitatory phase over the background phase.

The magnitude of the response for the excitatory phase increased as the zebrafish embryo developed from 24 hpf until 31 hpf. After 31 hpf, the excitatory response started to level off (Fig 1). A marked increase in the peak response (almost double) occurred from 30 hpf to 31 hpf, and there was noticeable change in the behavioral response of the dechorionated embryos. At 30 hpf, the embryos had stimulus-induced arrhythmic tail flexion movements, while at 31 hpf they had more rhythmic tail movements indicative of swimming motion, resulting in a higher movement score. For the background phase of the PMR test, the response steadily increased from 24 hpf to 32 hpf. The refractory phase remained unchanged as the embryos developed from 24 hpf to 32 hpf. The number of non-responding embryos decreased from 4.8% (29 embryos) to 0.3% (2 embryos) from 24 hpf to 30 hpf and remained at 0.3% at 31 hpf. At 32 hpf the number of non-responders increased slightly to 0.7% (4 embryos).

### Nanomaterial exposures

Of the 15 nanomaterials tested, 13 had positive behavioral response profiles (hypoactivity or hyperactivity) in the PMR test (Table 1). Concentrations above 50 μg/mL caused visual impairment of the PRAT imaging system with some of the nanomaterials, so Ethovision was unable to accurately track the zebrafish embryo in the well. In these cases, only behavioral differences that were statistically significant at concentrations ≤ 50 μg/mL are reported.

**Table 1:** 

Testing Material	PMR (by phase)			Highest Concentration Tested (μg/mL)	30 hpf LC50 (μg/mL)	30 hpf EC50 (μg/mL)	120 hpf LC50 (μg/mL)	120 hpf EC50 (μg/mL)
	Background (μg/mL)	Excitatory (μg/mL)	Refractory (μg/mL)					
30 nm Aluminum Oxide (alpha)	50	NA	None	500	66.3	ND	67.4	ND
10 nm Aluminum Oxide (gamma)	>50	>50	None	500	>500	ND	>500	ND
25 nm Cerium Oxide	>50	23.2	None	500	250	232	242	163
15–20 nm Iron Oxide	>50	23.2	None	500	>500	ND	>500	ND
20 nm Iron Oxide (gamma)	23.2	50	None	500	142	ND	155	ND
20 nm Silica	2.32*	None	None	500	>500	ND	>500	ND
50 nm Silica	2.32*	None	10.7*	500	>500	ND	>500	ND
Silver Nitrate	0.00232*	0.0108	None	50	1.01	0.15	0.08	0.37
5 nm Silver	0.5	0.5	0.108*	50	3.81	2.37	0.71	2.34
25 nm Silver	0.05	0.108	None	50	6.8	20	3.68	14.6
75 nm Silver	0.108	1.08	None	500	154.26	189	33.1	74.9
75 nm Silver (NIST)	0.232	0.5	None	50	ND	ND	43.3	ND
25 nm Titanium Oxide Nanopowder	None	5	None	500	378	ND	325	ND
5–15 nm Titanium Oxide (rutile)	50	>50	None	500	302	411	284	434
30–50 nm Titanium Oxide (anatase)	10.7	>50	None	500	>500	ND	>500	ND
30–50 nm Titanium Oxide (rutile)	>50	>50	None	500	>500	ND	>500	ND

Nanomaterial Effects on Zebrafish Embryos: PMR performed at 30 hpf, 30 hpf and 120 hpf mortality and morphological endpoints (LC50 and EC50). Lowest effect concentration shown for PMR tests with calculated p-value of less than 0.05. Nanomaterials that had a reported PMR of >50 μg/mL had observable behavioral effects but do to the turbidity of the wells at higher concentrations accurate detection with the tracking software could not be verified. Malformations were observed in only 0.007% of all the dechorionated control embryos.

Notes:

* = These PMR responses were hyperactive; all other PMR responses were hypoactive.

ND = These values could not be determined.

**Fig 2:**
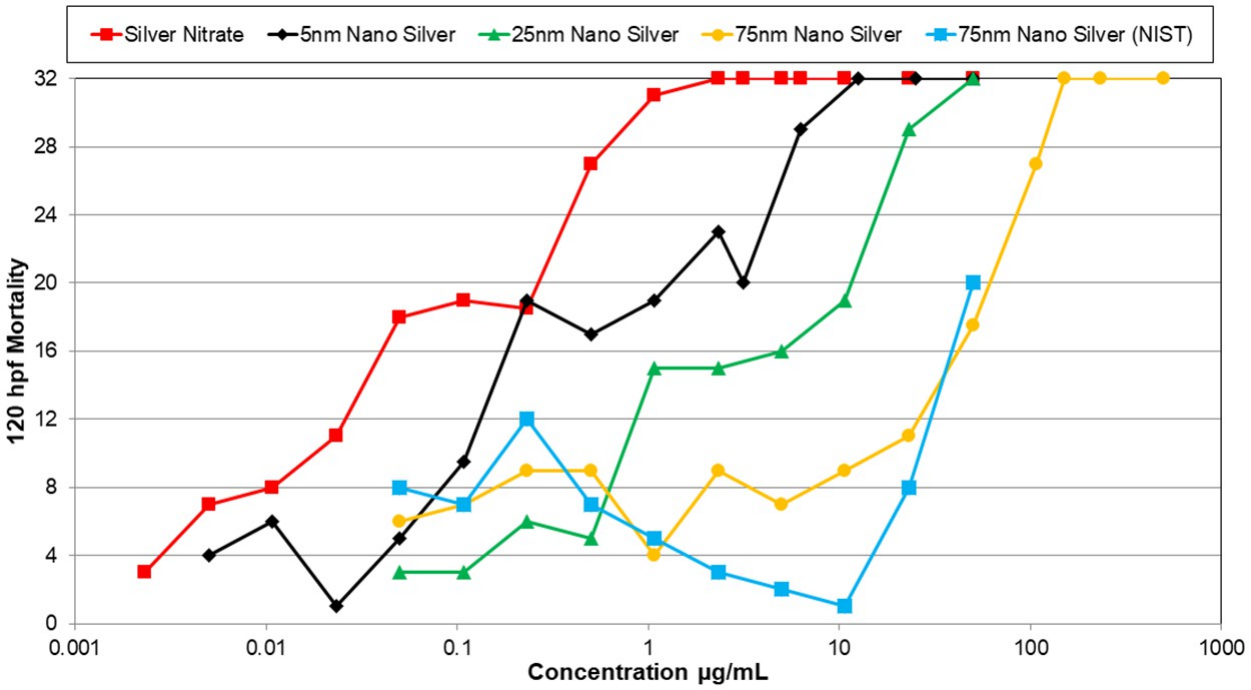
Mortality curves of zebrafish embryos exposed to different nanomaterials. 120 hpf mortality curves of zebrafish embryos exposed to different nanomaterials. Mortality curves consist of multiple tests. If the concentrations between tests of the same nanomaterial overlapped, the average mortality at the concentration was reported in the mortality curve. This figure shows the mortality curve of silver nanomaterials based on size without the concentrations being adjusted for the ionic concentration of silver in solution. (120 hpf (LC50) for the silver nanomaterials are as followed: silver nitrate (0.08 μg/mL), 5 nm nano silver (0.71 μg/mL), 25 nm nano silver (3.68 μg/mL), 75 nm nano silver (33.1 μg/mL), and 75 nm nano silver (NIST) (43.3 μg/mL)).

**Fig 3:**
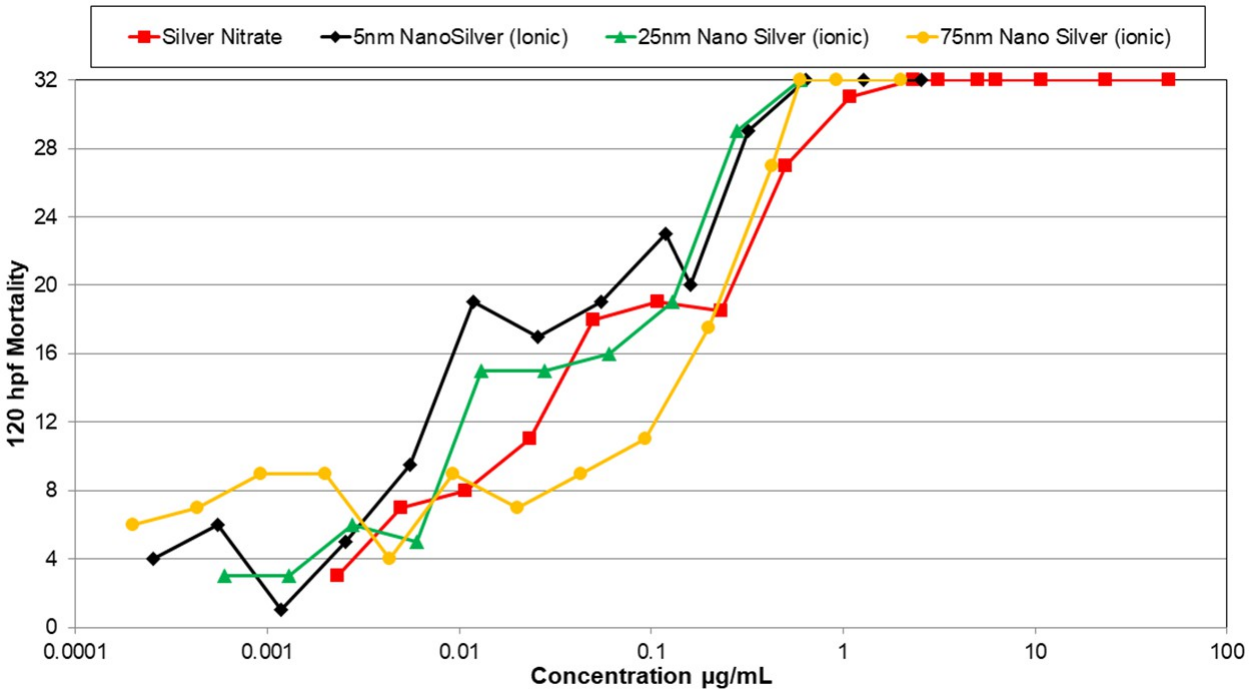
Mortality curves of zebrafish embryos exposed to different nanomaterials. 120 hpf mortality curves of zebrafish embryos exposed to different nanomaterials. Mortality curves consist of multiple tests. If the concentrations between tests of the same nanomaterial overlapped, the average mortality at the concentration was reported in the mortality curve. This figure shows the mortality curves of silver nanomaterials with the concentrations adjusted for the concentration of ionic silver in solution. (120 hpf (LC50) for the silver nanomaterials with adjusted concentrations are as followed: silver nitrate (0.08 μg/mL), 5 nm nano silver (0.03 μg/mL), 25 nm nano silver (0.04 μg/mL), and 75 nm nano silver (0.16 μg/mL)).

Silver nanomaterials were the most toxic nanomaterials tested, and 5 nm silver was the most toxic of these, with an LC50 of 0.71 μg/mL (Table 1). However, the 5 nm silver nanomaterial was much less toxic than the ionic silver (silver nitrate). Toxicity induced by the silver nanoparticles decreased with increasing nanoparticle size (Fig 2). However, when the concentrations of silver nanomaterials were adjusted for their ionic component using calculated dissolution rates (Table 2), the toxicity was relatively consistent across all sizes of the silver nanomaterials that were tested (Fig 3). Therefore, the toxicity observed by the nanomaterials is most likely related to the amount of free ionic silver in solution. Results from the behavioral testing (PMR) were consistent across different sized nanomaterials, with hypoactivity detected in the background and excitatory phases (Table 1). Only the 5 nm silver nanomaterial differed, in that hyperactivity was detected in the refractory phase at 0.108 μg/mL. Silver nitrate caused behavioral changes in the PMR at much lower concentrations than the silver nanomaterials, with a different response pattern: hyperactivity was detected in the background phase, hypoactivity was detected in the excitatory phase, and the refractory phase showed a normal response. Exposures to silver nanomaterials also resulted in delayed developmental progress in the zebrafish embryos.

**Table 2:** 

**Nanomaterial**	**Manufacturer Reported Values**			**Size**				**Concentration**		
	**Vendor, Lot Number**	**Particle Size (nm)**	**Concentration**	**Particle Diameter by TEM (nm)**	**Hydrodynamic Diameter by Batch-mode DLS (nm)**	**Ploydispersity Index by Batch-mode DLS**	**Hydrodynamic Diameter by Flow-mode DLS (nm)**	**Total [ion] by ICP-MS (mg/g)**	**Total [Compound] by ICP-MS**	**Total [Compound] by TGA**
Aluminum Oxide (alpha)	US Nano US7010	30	20% wt.	30 ± 17	194 ± 2	0.07 ± 0.01	Unsuccessful	79 ± 20	15% wt.	19% wt
Aluminum Oxide (gamma)	US Nano US7020	10	20% wt.	24 ± 7	122 ± 2	0.14 ± 0.01	Unsuccessful	53 ± 28	10% wt.	16% wt
Cerium Oxide	Sigma Aldrich 643009 (MKBW6379V)	<25	10% wt.	Aggregates/ Agglomerates	257 ± 11	0.15 ± 0.02	Not Tested	141 ± 42	17% wt.	20% wt.
Iron Oxide	US Nano US7568	15–20	20% wt.	12 ± 4	238 ± 15	0.25 ± 0.01	Major: 34–100 Minor: 100–260	68 ± 12	9% wt.	11% wt.
Iron Oxide (gamma)	US Nano US7558	20	10% wt.	Aggregation/ Agglomeration	242 ± 26	0.34 ± 0.02	Not Tested	107 ± 38	15% wt.	26% wt.
Silica Nanospheres	nanoComposix JEA0156 (MEL0010)	20 ± 4	10 mg/mL	23 ± 4	29 ± 0	0.11 ± 0.01	Major: 25–37 Minor: 174–184	-	-	4.0 mg/mL
Silica Nanospheres	nanoComposix JEA0088 (MEL0053)	50 ± 4	10 mg/mL	48 ± 5	58 ± 0	0.02 ± 0.01	Not Tested	-	-	9.1 mg/mL
Silver	nanoComposix KJW1980A (ECP1598)	5 ± 2	5 mg/mL	5 ± 1	14 ± 3	0.45 ± 0.02	Major: 14–23 Minor: 46–160	-	4.62 ± 0.05 mg/mL	7.8 mg/mL
Silver	nanoComposix CLF0494A (DAC1541)	25 ± 5	5 mg/mL	7 ± 2 24 ± 5	5 ± 0	0.56 ± 0.01	Major: 25–26 Minor: 12–15	-	4.51 ± 0.05 mg/mL	3.5 mg/mL
Silver	nanoComposix DMW0382 (ALJ0044)	75 ± 5	5 mg/mL	83 ± 9	121 ± 2	0.08 ± 0.02	103–139	-	4.49 ± 0.04 mg/mL	2.2 mg/mL
Silver	National Institute of Standards and Technology (NIST)	75	1 mg/mL	-	-	-	-	-	-	-
Titanium Oxide Nanopowder	ECONIK 614031098	25	-	-	-	-	-	-	-	-
Titanium Oxide (rutile)	US Nano US7050	5–15	15% wt.	63 ± 19	683 ± 209	0.29 ± 0.06	Major: 43–200 Minor: 285–320	72 ± 20	12% wt.	15% wt.
Titanium Oxide (anatase)	US Nano US7071	30–50	40% wt.	41 ± 14	219 ± 36	0.28 ± 0.03	Not Tested	231 ± 66	39% wt.	36% wt.
Titanium Oxide (rutile)	US Nano US7070	30–50	20% wt.	55 ± 15	780 ± 68	0.22 ± 0.03	Unsuccessful	42 ± 22	7% wt.	9% wt.
**Nanomaterial**	**Sterility and Endotoxin**		**Surface Characterization**				**Purity**	**Stability**		
	**Sterility (CFU/mg)**	**Endotoxin (EU/mg)**	**Coating Detected**	**Coating Identity**	**Coating Concentration by TGA (mass coating per mass NP)**	**Zeta Potential (mV)**	**Metal Impurities by ICP-MS**	**Free [ions] by ICP-MS (1)**	**Free [ions] by ICP-MS (2)**	**Free [ions] by ICP-MS (3)**
Aluminum Oxide (alpha)	Negative	<0.5	Possible	Unknown	5%	25 ± 2	Y, Zr, Hf	<0.1% (15 June 2016)	<0.1% (30 August 2016)	<0.3% (30 November 2016)
Aluminum Oxide (gamma)	Negative	<0.5	Possible	Unknown but different than US7010	23%	45 ± 1	Zn	0.1% (15 June 2016)	<0.1% (30 August 2016)	<0.1% (30 November 2016)
Cerium Oxide	Not Tested	Not Tested	-	-	-	18 ± 2	None	-	-	-
Iron Oxide	Negative	<0.05	Possible	Unknown	15%	-36 ± 0	Mn, Zn	0.8% (09 June 2016)	1.2% (26 August 2016)	0.3% (21 November 2016)
Iron Oxide (gamma)	Bacteria 72,000	6170	Possible	Unknown	17%	-36 ± 1	Mn, Zn	2.5% (09 June 2016)	1.8% (26 August 2016)	1.8% (21 November 2016)
Silica Nanospheres	Negative	3.1	Possible	Unknown	13%	-27 ± 1	-	-	-	-
Silica Nanospheres	Mold	<0.005	Possible	Unknown	19%	-53 ± 4	-	-	-	-
Silver	Negative	1.9	Yes	Polyvinylpyrrolidone (per manufacturer; size unknown)	29%	-33 ± 0	Zn	5.1% (01 June 2016)	18% (31 August 2016)	-
Silver	Negative	<5	Yes	Polyvinylpyrrolidone (per manufacturer; size unknown)	5%	-20 ± 1	Zn, Cu	1.2% (01 June 2016)	13% (31 August 2016)	-
Silver	Negative	2.5	Yes	Polyvinylpyrrolidone (per manufacturer; size unknown)	15%	-24 ± 1	Zn	0.4% (01 June 2016)	2.5% (31 August 2016)	-
Silver	-	-	-	-	-	-	-	-	-	-
Titanium Oxide Nanopowder	-	-	-	-	-	-	-	-	-	-
Titanium Oxide (rutile)	310	28	Possible	Unknown	10%	-20 ± 1	Zn, Hf	0% (28 September 2016)	<0.1% (29 November 2016)	-
Titanium Oxide (anatase)	Negative	<0.5	Possible	Unknown	10%	-12 ± 0	V, Mn, Fe, Zn, Sr, Ce, Pb, U	0% (28 September 2016)	<0.1% (29 November 2016)	-
Titanium Oxide (rutile)	Negative	<0.05	Possible	Unknown	1%	7 ± 1	Zn,Zr,Sn	0% (28 September 2016)	0.1% (29 November 2016)	-

Nanomaterial physico-chemical data. Physical chemical data obtained from the NCL’s final report on the nanomaterials. For nanomaterials that have two lot numbers the lot number that is in parentheses and is in bold face was the lot that was tested for toxicity testing but was not tested by the NCL for its physical chemical properties. Nanomaterials that did not have a concentration listed for the section "manufacturer reported values" were in powder form and prepared following the manufacturer’s instructions.

The 25 nm cerium oxide nanomaterial had an LC50 of 242 μg/mL and caused behavioral effects in the PMR test; exposed embryos had reduced movement during the excitatory phase. The cerium oxide nanomaterial settled rapidly in solution. Analysis of a different lot than the one used here showed that this nanomaterial forms aggregates and/or agglomerates in solution which makes their hydrodynamic size large; the measured size of the cerium oxide (257 ± 11 nm by DLS) was more than ten times larger than what was reported by the manufacturer (25nm) [21].

Aluminum oxide nanomaterial toxicity varied with particle size. The 30 nm aluminum oxide (alpha) nanomaterial was found to be more toxic than the smaller 10 nm aluminum oxide (gamma) (Table 1) and caused hypoactivity at 50 μg/mL; the 10 nm aluminum oxide (gamma) caused no behavioral effects in the PMR test. The structure of the two nanomaterials differed as well: the 10 nm aluminum oxide nanomaterials had a rod-like structure, while the 30 nm aluminum oxide nanomaterials had flake-like structure. Surface coatings were found on both aluminum oxide nanomaterials; the coating identity is unknown, but differs between the two types.

The titanium oxide nanomaterials tested had low toxicity (Table 1). An LC50 could not be determined for the larger 30–50 nm titanium oxide anatase and rutile nanomaterial due to low mortality. Only the 25 nm titanium oxide nanopowder caused behavioral (hypoactive) responses during the background phase of the PMR. The measured size of the 5–15 nm titanium oxide (rutile) was much larger than the nominal size (63 ± 19 nm). All of the titanium oxide nanomaterials had possible coatings of unknown composition (Table 2). The titanium oxide nanomaterials settled rapidly in solution, especially the 30–50 nm titanium oxide (rutile).

The iron oxide nanomaterial caused behavioral effects in the PMR test. The 15–20 nm iron oxide (gamma) reduced activity (hypoactive) in the excitatory phase. The smaller 15–20 nm iron oxide (gamma) was not very toxic, with an LC50 >500 μg/mL.

The silica nanospheres tested were relatively non-toxic, with LC50s above the highest concentration tested (500 μg/mL). Malformation rates did not exceed 5% in any of the tested concentrations. However, the PMR test showed hyperactivity in the background phase for both the 20 nm and 50 nm silica nanospheres at 2.32 μg/mL. This behavioral response profile was unique among the nanomaterials tested.

The toxicities of the 20nm iron oxide and the 5-15nm titanium oxide (rutile) nanomaterials are not discussed as part of their nanomaterial sets because they were contaminated by bacteria and endotoxin (Table 2), and it could not be determined whether the observed toxicity is related to the nanoparticle itself or to the contamination. The 20 nm iron oxide (gamma) had an LC50 of 155 μg/mL and was found to form aggregates and/or agglomerates. It caused reduced activity (hypoactive in the excitatory and background phases). The 5–15 nm titanium oxide (rutile) nanomaterial had an LC50 of 302 μg/mL and caused behavioral (hypoactive) responses during the background phase of the PMR. Zebrafish embryos exposed to 5–15 nm titanium oxide also showed delayed developmental progression. The measured size of this nanomaterial (63 ± 19 nm) was much larger than its nominal size. The increased size might be due to aggregation or agglomeration of the nanomaterials, as occurred with the cerium oxide nanomaterial.

## Discussion

### PMR time series test

The time series evaluation of PMR behavioral responses showed that zebrafish embryo developmental stage has a major effect on the magnitude of observed response. It is known that the PMR is first detectable at 24 hpf, continuing until 40 hpf [12, 13, 23, 24, 28, 29] and Kokel et al. (2013) [12] found that the PMR increases with developmental age. Kokel et al. (2013) also found during the PMR behavioral test that 27 hpf zebrafish embryos and younger failed to be triggered to move by a light stimulus during the excitation phase, rather the first exposure to a white light flash caused inactivity [12]. Do to the large amount of variability of information in the literature we decided to do an optimization of the PMR response before we conducted the study to ensure a repeatable and consistent response. To our knowledge, this is the first time data from all three phases of the PMR has been used to determine the time of maximum response in this behavioral assay. Our data show that 31 hpf has the highest magnitude PMR response with the lowest number of non-responding embryos. The response to light stimuli at 27 hpf causes a statistically significant increase in movement during the excitatory phase which is slightly earlier than previously reported in Kokel et al. (2013) [12]. We chose a 30 hpf time point for this test since it provided a greater response than the 24 hpf time point used by others, and it was a better fit with our laboratory work flow, while still providing a low percentage of non-responding embryos (0.3%). We conclude that the zebrafish PMR behavioral assay can be improved by changing the test duration from 24 hpf to either 30 or 31 hpf to maximize response in the excitatory phase.

### Nanomaterial exposures

Silver nanomaterial toxicity that was likely related to the amount of ionic silver that was released via oxidative dissolution. When silver nanoparticle concentrations were expressed as the estimated concentration of ionic silver in solution, the concentration-response relationship was close to that of the embryos exposed to silver nitrate alone. The 75 nm silver nanomaterial had a higher mortality rate than the 75 nm silver nanomaterial National Institute of Standards and Technology (NIST) standard at 30 hpf, one possible explanation for this could be there was more ionic silver in solution for the 75 nm silver nanomaterial than the NIST standard. For future studies involving toxicity testing of silver nanomaterials, the ionic component of the solution should be determined to help distinguish toxicity related to the parent nanomaterial from that resulting from the ionic component. These toxicity results are consistent with other studies conducted on silver nanomaterials [17, 26, 30–32]. All silver nanomaterials tested showed effects in the PMR assay, mainly hypoactive responses. These PMR responses may be indicative of neurotoxicity, as found in previous studies [33, 34].

The cerium oxide LC50 of 242 μg/mL is consistent with van Hoecke et al. (2009) [35], who reported an LC50 of >200 mg/L and found that cerium oxide nanomaterials aggregated on the zebrafish chorion. To our knowledge, this is the first time that potential neurological impacts of cerium oxide nanomaterials has been shown in PMR testing; there was decreased movement in response to the white light stimulus in the excitatory phase. Cerium oxide also caused caudal fin malformations at higher concentrations (≥150 μg/mL).

The 25 nm titanium oxide nanopowder was found to be neuroactive, causing reduced movement in the PMR assay. This result adds to the growing body of work indicating that these nanomaterials can cause behavioral effects, specifically reduced movement [36, 37].

The endotoxin and sterility testing found contamination in two of the nanomaterials tested, iron oxide and titanium oxide (Table 2). The 20 nm iron oxide contained 72,000 bacterial colony forming units (CFU) and 6,170 Endotoxin Units (EU) per mg, which could have contributed to the toxicity that was observed. The most toxic of the titanium oxide nanomaterials tested, the 5–15 nm titanium oxide (rutile), was the smallest of titanium oxide nanomaterials, but also had 310 CFU/mg and 28 EU/mg, which may have increased toxicity. Bacterial and endotoxin contamination of nanomaterials is not uncommon and may affect toxicological findings [38, 39]. Thus, further exploration of the effects of bacterial and endotoxin contamination on nanomaterial toxicity may be warranted.

Zebrafish larva exposed to both 20 nm and 50 nm silica nanospheres had hyperactive responses during the background phase; no other nanomaterial tested showed this response. While Pham et al. (2016) [14] did not find hyperactivity during the background phase of the PMR, they found that 20 and 50 nm silica nanomaterials caused hypoactivity during the excitatory phase and that the 80 nm silica nanospheres caused hyperactivity during the excitatory phase. However, Pham et al. used zebrafish embryos with their chorions intact during the exposures, so the chorion may have acted as a barrier to silica nanoparticle exposure [17]. Previously published work has shown that the dechorionated zebrafish embryo model may be more sensitive to toxicants than the standard chorionated zebrafish embryo model [40]. Dechorionation prior to zebrafish embryo testing removes any potential barrier effect the chorion may have on the uptake of the nanomaterial. In addition, automated dechorionation is an effective and reproducible method to remove the chorions from zebrafish embryo. Previous manual dechorionation methods using pronase with manual agitation to dechorionate is highly variable and dependent upon the technician preforming the procedure. Henn and Braunbeck (2011) reported zebrafish embryos manually dechorionated with pronase at 6 hpf had a survival rate as low as 20%. [20] Using an automated dechorionation system has improved the process of using pronase to dechorionate zebrafish embryos at early developmental ages by removing the technician to technician procedural variability. Thus, automated dechorionation is a valuable addition to the assay by consistently removing the chorion with high reproducibility, results in high survival rates and low malformation rates, and also removes any potential barrier effect of the chorion on the uptake of the nanomaterials into the embryo.

Application of this model is limited to those nanomaterials that can be readily made in aqueous solutions; given potential lot to lot variability between nanomaterials, physical and chemical characterization is critical. Another potential limitation of the study is that only the nominal waterborne concentration of the nanomaterials in the well was reported. This means comparisons between the bioavailability and the real measured concentration of the nanomaterials in the well could not be made. While the nanomaterials that were found to be contaminated and contained endotoxins within their respective group had higher toxicity than non-contaminated nanomaterials, additional testing is needed to confirm the relationship between bacterial and endotoxin contamination and toxicity. Nevertheless, sterility and endotoxin testing may be helpful in toxicity test interpretation. Physicochemical nanomaterial characterization also highlighted discrepancies in the manufacturer reported and measured sizes of some of the nanomaterials, specifically the 5–15 nm titanium oxide (rutile) and the cerium oxide nanomaterials. Determination of the presence or absence of coatings on nanomaterials and their composition is also valuable, as coatings may also alter the toxicity of the nanomaterial. These results reinforce the need for physical and chemical characterization of nanomaterials use in toxicity testing. The zebrafish PMR test using automated dechorionation provides high throughput toxicity testing to rapidly assess the potential toxicity profile of newly manufactured nanomaterials. As a rapid screen it can help to quickly identify potential toxicity issues with nanomaterial formulations so that additional physical chemical and toxicity studies may be conducted to help determine the potential environmental and human health risks of these new materials.

## Supporting information

S1 Table Zebrafish morphological endpoints. This table shows all the endpoints that were recorded for each test at 30hpf (early morphological endpoints) and 120hpf (late morphological endpoints). Abbreviations for the endpoints are on the left side of the table with the corresponding descriptor on the right side.
(DOCX)
