# Supplementary material for: High throughput embryonic zebrafish test with automated dechorionation to evaluate nanomaterial toxicity
Source: PLoS One. 2022 Sep 16;17(9):e0274011. doi: 10.1371/journal.pone.0274011 (PMC9481008; doi:10.1371/journal.pone.0274011)
Supplement: S1 Table — This table shows all the endpoints that were recorded for each test at 30hpf (early morphological endpoints) and 120hpf (late morphological endpoints). Abbreviations for the endpoints are on the left side of the table with the corresponding descriptor on the right side. (DOCX) [file pone.0274011.s001.docx]

| **Endpoints Recorded at 30hpf and 120hpf (Key)** | |
| --- | --- |
| MO | Mortality at 30hpf |
| DP | Delayed Development at 30hpf |
| NC | Notochord Malformation at 30hpf |
| MORT | Mortality at 120hpf |
| YSE | Yolk Sac Edema |
| AXIS | Axis Malformation |
| EYE | Eye Malformation |
| SNOU | Snout Malformation |
| JAW | Jaw Malformation |
| OTIC | Otic Vesicle(s) Malformation(s) |
| PE | Pericardial Edema |
| BRAI | Brain Malformation |
| SOMI | Somite Malformation |
| PFIN | Pectoral Fin Malformation |
| CFIN | Caudal Fin Malformation |
| PIG | Over Pigmentation or Lack of Pigmentation |
| CIRC | Lack of Circulation |
| TRUN | Trunk Malformation |
| SWIM | Swim Bladder Malformation |
| NC | Notochord Malformation |
| TR | Touch Response Deficiency |
| DNC | Do Not Count |
